# Inclusion of Ora-Pro-Nóbis (*Pereskia aculeata*) Leaf Meal in the Diet of Adult Nile Tilapia Improves Growth Performance and Intestinal Absorption Capacity Without Compromising Metabolic and Hematological Variables

**DOI:** 10.3390/vetsci12010015

**Published:** 2025-01-01

**Authors:** Émerson J. A. Matos, Jailson Novodworski, Rafaela M. Gonçalves, Elisabeth C. Urbinati, Robie A. Bombardelli, Fábio Meurer

**Affiliations:** 1 Graduate Program in Animal Science, Federal University of Paraná, 1540 Rua dos Funcionários, Cabral, Curitiba 80035-050, PR, Brazil; jnjailson@ufpr.br (J.N.); rafaela.mocochinski@ufpr.br (R.M.G.); fabiomeurer@ufpr.br (F.M.); 2 Department of Animal Morphology and Physiology, School of Agricultural and Veterinarian Sciences, São Paulo State University, Jaboticabal 14884-900, SP, Brazil; elisabeth.criscuolo-urbinati@unesp.br; 3 Center for Engineering and Exact Sciences, Western Paraná State University, Toledo 85903-000, PR, Brazil; rabombardelli@gmail.com

**Keywords:** alternative feed, fatty liver degeneration, feed conversion ratio, intestinal villi, tilapia farming

## Abstract

Corn and soybeans are commodities and ingredients of global interest, whose prices fluctuate based on global demands. In this sense, this study aimed to assess ora-pro-nóbis (*Pereskia aculeata)* leaf meal (OLM) as an alternative to be included in the diets of Nile tilapia (*Oreochromis niloticus*). The optimal inclusion level of OLM in tilapia diets is investigated herein, aiming to improve their growth performance and health. Five diet variations containing OLM (0%, 5%, 10%, 15%, and 20%) were tested. Feed conversion and protein efficiency rates in the 5% and 10% OLM groups were statistically similar to the control group (*p* ≤ 0.05) and lower in the 15% and 20% OLM groups. Fish fed 5% and 10% OLM diets showed better feed efficiency, while higher OLM levels (15% and 20%) led to reduced carcass protein and ether extract levels. Increasing OLM levels enhanced intestinal villi height and area, associated with improved nutrient absorption and decreased liver fat degeneration, suggesting dietary adaptation and healthier liver conditions. Thus, OLM can be included up to 10% in tilapia diets, improving their growth performance, feed efficiency, and intestinal absorptive capacity without adversely affecting other parameters.

## Simple Summary

This study assessed the effects of adding ora-pro-nóbis (*Pereskia aculeata*) leaf meal (OLM) to Nile tilapia diets during their growth phase, aiming to identify potential health and growth benefits. Diets containing 0%, 5%, 10%, 15%, and 20% OLM were tested, and the findings demonstrate that the 5% and 10% OLM options resulted in similar feed conversion and protein efficiency rates compared the control group while also leading to better feed efficiency. The diets containing higher OLM levels (15% and 20%), however, slightly reduced carcass protein and fat levels. Additionally, increasing OLM levels enhanced intestinal villi height and area, improving nutrient absorption, and reduced liver fat, suggesting better liver health. Therefore, diets containing up to 10% OLM can be safely included in tilapia diets, with no negative growth performance, health, feed efficiency, or nutrient absorption effects.

## 1. Introduction

Food security has become a global challenge [1], making the use and preservation of natural resources paramount. According to the Food and Agriculture Organization (FAO) [2], aquatic food production will increase by 15% by 2030 and must consider the health of aquatic ecosystems, pollution prevention, biodiversity protection, and social equality, a movement called “Blue Transformation”.

In this sense, aquaculture should avoid the use of animal-based meals such as fishmeal due to both the high ingredient costs and decreased market availability [3]. According to Farhad et al. [4], the use of fishmeal negatively affects wild fish populations, as this product is produced from wild-caught fish. In addition, animal-based ingredients contain higher amounts of nitrogen and phosphorus compared to plant sources, which can increase water body eutrophication [5].

In place of fishmeal and oil, plant ingredients such as soybean by-products, maize, and wheat have become conventional ingredients in aquaculture activities [6]. However, competition between aquaculture and animal feed industries has increased for these feed ingredients [7]. Additionally, both the aquaculture sector and demands for aquafeed are growing steadily. Therefore, the selection of feed ingredients that can be produced sustainably and can grow with these industry sectors has become paramount.

Due to their availability, leafy meals, abundant throughout the year in the tropics, are gaining significant attention from researchers as a source of nutrients, energy, minerals, vitamins, and bioactive compounds useful in improving growth, feed efficiency, and fish health [8,9]. Research in this area has contributed to the production of more economically sustainable, environmentally friendly, and viable fish feeds.

Ora-pro-nóbis (*Pereskia aculeata*) is an unconventional food plant (UFP) containing high proteins, minerals, and carotenoids contents, with its leaves representing excellent sources of these nutrients [10]. Some studies indicate that ora-pro-nóbis leaf meal (OLM) contains from 16.94% to 28.99% crude protein and between 5.07% and 5.81% ether extract [11,12]. In addition to their nutritional value, ora-pro-nóbis leaves can be harvested several times throughout the year, increasing the availability of leaf meal.

Ora-pro-nóbis is easily propagated as its reproduction can be carried out asexually through cutting, with seedlings that can be produced in bags or planted directly in the soil [13]. Its average productivity in dense planting with successive pruning was studied by Souza et al. [14], who reported eight consecutive harvests over 12 months, with a planting density of 10 plants per m^2^ considered optimal, yielding fresh and dry leaf matters of 144 t ha^−1^ year^−1^ and 25.6 t ha^−1^ year^−1^, respectively. Ora-pro-nóbis leaf meal, in turn, is produced by grinding the dry biomass of this plant, composed of leaves and petioles. Under the same cultivation conditions (~10 plants per m^2^), ora-pro-nóbis can yield 5759 kg of leaf protein per hectare annually. In comparison, the annual protein production per hectare from soybeans (*Glycine max* (L.) *Merr*.) and corn (*Zea mays* L.), considering only the grains, averages much lower values of 1154 kg and 514.7 kg, respectively [14].

The use of OLM in animal nutrition, however, is still relatively recent. Souza et al. [15] evaluated the replacement of wheat bran by OLM in broiler chicken diets and reported that an inclusion level of 10% resulted in better weight gains compared to the control treatments (0%) and 5% replacement treatments.

Tilapia *(Oreochromis niloticus*) is the most cultivated fish species in Brazil and ranks among the most widely farmed globally. Recent data indicate that it accounted for 65% of Brazilian fish production in 2023 [16]. This species stands out for its hardiness, adaptability to adverse environmental conditions, and rapid growth, and research has demonstrated that incorporating leaf meal can partially replace conventional ingredients in diets for Nile tilapia without negatively impacting fish growth [17,18].

No studies evaluating levels of OLM inclusion as a dietary ingredient in the formulation of fish feeds, including levels appropriate for fish health, are available to date. In this context, this study aimed to assess the effect of incorporating OLM into the diet of Nile tilapia on growth performance, body chemical composition, metabolic/enzymatic, hematological variables, and intestinal histology.

## 2. Materials and Methods

### 2.1. Location and Experimental Design

This study was conducted at the Aquaculture Technology Laboratory (LATAq) be longing to the Federal University of Paraná (UFPR), Advanced of Jandaia do Sul Campus, Paraná, Brazil. A total of 150 Nile tilapia adults (287.85 ± 11.26 g) were distributed in 15 cages (0.3 m^3^) in a completely randomized design, comprising 10 fish per experimental unit (cage), for an experimental period of 98 days. The experimental units were distributed in a recirculation system composed of two geomembrane tanks (30,000 m^3^), one of which contained the experimental units and the other biological media and aquatic plants functioning as the system’s biofilter.

### 2.2. Feed Ingredients and Experimental Diets

The OLM was prepared from locally grown ora-pro-nóbis leaves during the summer/autumn of 2021. The leaves were dried in a forced ventilation oven for 72 h at 55 °C, crushed using a 0.5 mm sieve, and stored chilled in a refrigerator in plastic bags. Based on the OLM dry matter composition (Table 1) and digestibility values [19], OLM was incorporated to form four of the five experimental tilapia diets (Table 2).

Isoprotein and isoenergetic diets, concerning digestible protein and energy, respectively, were prepared by adding 5%, 10%, 15%, and 20% OLM, while the control diet contained no OLM (0%). In addition to the digestible OLM values indicated by Matos et al. [19], the OLM inclusion level selection considered compliance with the nutritional Nile tilapia requirements as described by Carneiro et al. [20]. Consequently, 20% was established as the highest possible inclusion level based on these requirements. From there, inclusion levels were defined in decreasing increments, ranging from 20% to 5%.

The ground ingredients were extruded to a pellet sizes of 3.0 mm using an extruder (Exteec^®^ Máquinas, Ribeirão Preto, SP, Brazil) and dried in a forced recirculation oven for 48 h at 55 °C. The diets were stored at 8 °C until use. Feeding management included three daily feedings until apparent satiety (at 8:30 AM, 1:30 PM, and 5:30 PM), with the amount of feed limited to 1.5% of the average fish biomass.

### 2.3. Growth Performance Analysis and Body Yields

At the end of the experimental period, all fish were anesthetized by immersion in anesthetic solution (Eugenol) and then counted, measured, and weighed to assess average weight gain (WG), daily weight gain (DWG), final weight (FW), daily feed intake (DFI), feed conversion ratio (FCR), protein efficiency rate (PER), feed efficiency (FE), and survival rate (SR), according to Carneiro et al. [20]

Where:

WG=(final average weight−initial average weight);

$$DFI(g)=\frac{feed\;intake}{experimental\;days};$$

$$FCR=\frac{{feed\;intake}_{\left(g\right)}}{{weight\;gain}_{\left(g\right)}\;};$$

$$PER=\frac{{weight\;gain}_{\left(g\right)}\;}{{\;ingested\;protein}_{\left(g\right)}};$$

$$FE=\frac{weight\;gain}{feed\;ingested};$$

$${SR}_{\left(\%\right)}=\left(\frac{final\;number\;of\;collected\;fish\;}{initial\;number\;of\;stocked\;fish\;}\right)*100.$$

Ten fish from each experimental unit were euthanized using a solution containing excess anesthetic to assess body yield parameters and subsequent chemical composition. Of these, three were frozen whole for body chemical composition analyses. Another four were used to assess body yields with and without the heads, and only samples with the heads were frozen for subsequent chemical composition analyses. The remaining three fish were utilized to determine fillet yields and the hepatosomatic, viscerosomatic, and visceral fat indices. The fillets were frozen for the chemical composition analyses, while the liver and intestine were preserved for histological evaluations. Fish were randomly selected for each analysis.

Other indices were also calculated, as follows:

$${CYH}_{\left(\%\right)}=\left(\frac{carcass\;weight\;with\;head}{weight\;of\;whole\;fish}\right)*100;$$

$${CYWH}_{\left(\%\right)}=\left(\frac{carcass\;weight\;without\;head}{fish\;weight\;}\right)\;*100;$$

$${FY}_{\left(\%\right)}=\left(\frac{fillet\;weight}{carcass\;weight}\right)\;*100;$$

$${HSI}_{\left(\%\right)}=\left(\frac{hepatopancreas\;weight}{body\;weight}\right)*100;$$

$${VSI}_{\left(\%\right)}=\left(\frac{viscera\;weight}{body\;weight}\right)\;*100;$$

$${VFI}_{\left(\%\right)}=\left(\frac{visceral\;fat\;weight}{body\;weight}\right)\;*100$$

### 2.4. Chemical Composition Analyses

Fillets, carcasses, and whole fish were identified and stored at −20 °C. The day before processing, the samples were placed in a refrigerator for slow thawing. The next day, still slightly frozen with firm tissues, they were chopped using a bandsaw slicer (1/3 CV) and processed using a 6 L cutter (model SPL-201). The resulting meat gel was maintained in an air recirculation oven for 72 h at 105 °C. After the drying process, the samples were crushed two more times, the first using an industrial blender and the second, a grinder, to reach the desired particle size for analysis (powder).

The crude protein analysis was performed using the micro-Kjeldahl distillation method, following AOAC methodologies [21]. The nitrogen content of each sample was multiplied by a conversion factor (6.25) to estimate crude protein (CP; method no. 984.13) values. Dry matter and mineral matter were also analyzed according to AOAC methodologies [21], where dry matter was determined by pre-weighing and subsequent drying in a hot air oven (DM; method no. 930.15), and mineral matter was obtained by incinerating the samples in a muffle furnace (MM; method no. 924.05). The ether extract of the samples was obtained using a Goldfish apparatus (Tecnal Ltda, Piracicaba, SP, Brazil) with hot solvent extraction (petroleum ether, 30–70 °C). Gross diet and fish energies were determined using an adiabatic bomb calorimeter.

The CP, DM, MM, EE, and GE of each diet were analyzed, employing the previously described methodologies. Fiber analyses were conducted according to official methods outlined by AOAC [21]. Crude fiber was determined by method no. 962.09, while neutral detergent fiber (NDF) and acid detergent fiber (ADF) were analyzed according to methods no. 2002.04 and no. 973.18, respectively. Lignin quantification was carried out through the specific procedure detailed in method no. 973.18.

### 2.5. Hematological Analyses

Five fish from each experimental unit were randomly selected and subjected to blood collection by caudal vein puncture. Blood samples were used to determine the number of erythrocytes employing a Neubauer chamber and hemoglobin concentrations, by a colorimetric method (Labtest^®^ kit, Lagoa Santa, MG, Brazil, Ref. 43). Then, mean corpuscular hemoglobin values were calculated as HMC = (hemoglobin rate *×* 10)/number of erythrocytes [22]. With the sampled blood, blood count extensions (two per animal) were carried out, comprising differential leukocyte counts (number of lymphocytes, neutrophils, monocytes, and eosinophils). The blood samples were stained in panoptic rapid dye for hematological use.

### 2.6. Metabolic and Enzymatic Analyses

A blood aliquot of each fish was centrifuged at 3000 rpm for 10 min, and serum aliquots were separated to determine total proteins (Ref. 418), albumin (Ref. 419), creatinine (Ref. 435), triglycerides (Ref. 459E), glucose (Ref. 112E), cholesterol (Ref. 460), calcium (Ref. 448M), and alkaline phosphatase (Ref. 440). Liver tissue fragments were frozen at −80 °C to quantify the activity of aspartate aminotransferase (Ref. 421) and alanine aminotransferase (Ref. 422) using commercial Gold Analisa Diagnóstica^®^ kits (Belo Horizonte, MG, Brazil). All analyses were performed on a spectrophotometer (Multiskan GO Microplate Reader—Thermo Fisher Scientific, Porto Salvo, Lisboa, Oeiras, Portugal).

### 2.7. Histological Analyses

#### 2.7.1. Intestinal Morphology

Fragments of the proximal intestine portions were removed, opened longitudinally, fixed on carton paper, washed with a 0.6% saline solution, and fixed in 10% buffered formalin for 24 h, for subsequent paraffin inclusion. The serial sections (3 μm thick) were stained with Hematoxylin and Floxin and Periodic Acid–Schiff (PAS) and Alcian Blue (AB) for photographic recording, followed by image analysis using the ImageJ software (version 1.54m) [23], to determine villi height and width (μm). In addition, goblet cells and intraepithelial leukocyte infiltration (ILI) were quantified (in un-units) by counting the goblet cells and leukocytes present in each of the photographed fields, with the aid of the aforementioned software. The results are presented as graphs.

#### 2.7.2. Liver Morphology

Liver fragments were fixed in 10% buffered formalin for 24 h and prepared for paraffin inclusion and subsequent staining with hematoxylin–eosin. The sections were analyzed under a white light microscope for the observation and description of the presence or absence of melanomacrophages and tissue inflammation. In addition, fat degeneration percentages were evaluated according to the degree of fat globules in hepatocytes. To this end, the following score was applied according to the occurrence/distribution of each alteration per slide: 0—no degeneration; 1—mild (<30%); 2—moderate (>30% and <70%), and 3—intense (>70%). An average degeneration degree was calculated using these score values for each experimental unit, classified as mild (0.1 to 1.0), moderate (1.1 to 2.0), and intense (2.1 to 3.0). This analysis followed an adaptation of the methods employed by Albinati et al. [24] and Albinati et al. [25].

### 2.8. Water Quality

Water quality parameters, i.e., water temperature, dissolved oxygen concentrations (DOCs), and pH, were determined using a multiparameter digital meter (Model AK-88, Akso Ltda, São Leopoldo, RS, Brazil), the first two measured three times a day with each feeding and the latter once a week. The water temperature, DOCs, and pH ranges were 22–28 °C, 5.2–8.1 mg/L, and 7.3–8.4, respectively, during the entire experimental period, all appropriate for tilapia farming [26].

### 2.9. Statistical Analysis

Data on productive growth performance, body chemical composition, metabolic/enzymatic, hematological, and intestinal histology variables were subjected to an Analysis of Variance (one-way ANOVA). When significant differences among the means were detected (*p* ≤ 0.05), Duncan’s test was applied, and the results are presented as graphs employing means and standard deviations.

## 3. Results

Growth performance parameters, represented by final weight, average weight gain, daily weight gain, daily feed intake, carcass yield with head, carcass yield without the head, and fillet yield, were not affected by OLM inclusion. However, the best apparent feed conversion ratios were observed in the diets containing 5% and 10% OLM inclusion, compared to 15% and 20%, while the control treatment showed similar results to both. Treatments with the highest levels of OLM inclusion (15% and 20%) exhibited a lower protein efficiency ratio (PER) compared to the other treatments. Concerning feed efficiency (FE), the highest averages were observed at OLM inclusion levels of 5% and 10%, which were similar to each other and superior to the other OLM treatments.

The hepatosomatic, viscerasomatic, and visceral fat indices, as well as survival percentages, were not significantly different (*p* > 0.05) among treatments (Table 3).

The approximate body chemical composition is depicted in Table 4. The moisture contents, crude protein, ether extract, and mineral matter of the fillets and the whole fish were not affected by diet composition (*p* > 0.05). An increase in moisture content (*p* ≤ 0.05) was observed, with a decrease in protein and body lipid carcass values at higher OLM inclusion levels. Mineral matter content was not altered in fillets, carcasses, or whole fish (Table 4).

The highest mean values of erythrocytes and hemoglobin values were observed in the control treatment (T0%), both significantly higher than those at the T10% and T20% OLM inclusions (*p* > 0.05). The mean corpuscular hemoglobin index and lymphocyte, neutrophil, monocyte, and eosinophil percentages were not significantly different among the experimental diets (Table 5). No significant changes (*p* ≤ 0.05) were observed in plasma variables or in liver enzymes (ALT and AST) in fish from treatments with up to 20% dietary OLM inclusion (Table 6).

**Figure 1:**
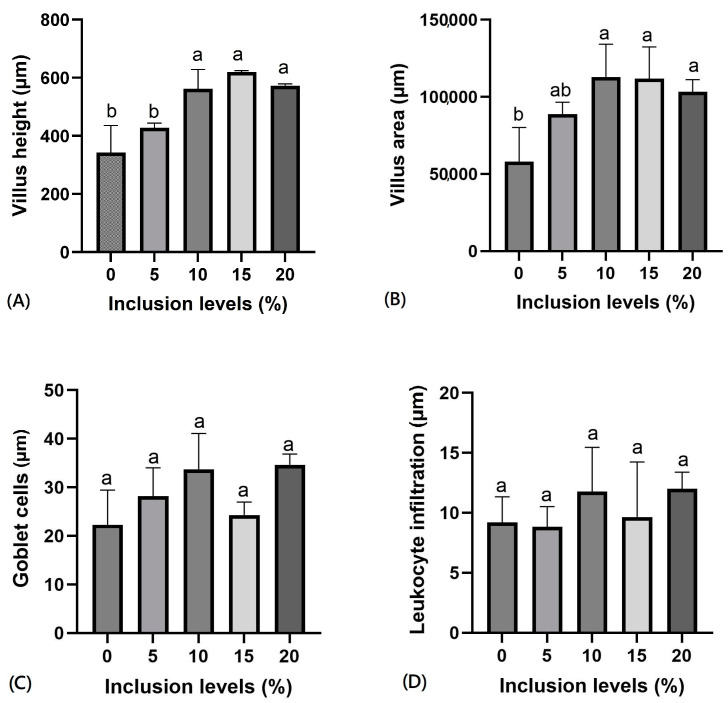
Intestinal morphology of Nile tilapia fed diets containing different levels of ora-pro-nóbis meal inclusion: (**A**) villi height; (**B**) villus area; (**C**) number of goblet cells; (**D**) number of leukocyte infiltrations. Different letters above the error bar indicate significant differences (*p* ≤ 0.05) from each other according to Duncan’s test. Values are expressed as means ± standard deviation.

**Figure 2:**
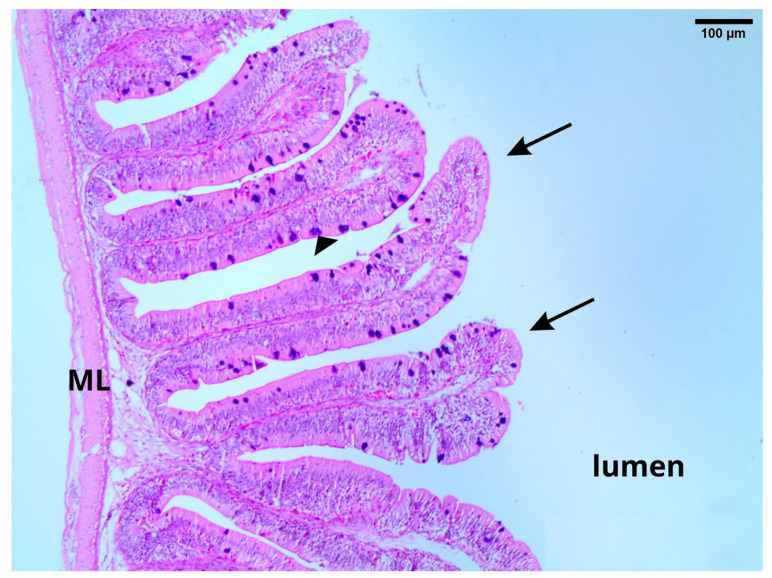
Photomicrograph of the small intestine of *O. niloticus.* Cross-section of the small intestine of tilapia showing the general organization, with the mucus layer in the form of villi (arrows). The numerous small goblet cells (arrowhead) are seen in the columnar epithelium lining the intestine in the previous plate stain purple with PAS/AB stain. Smooth muscle layer (ML).

Intestinal villi height and area in Nile tilapia increased significantly (*p* ≤ 0.05) with the increase in OLM (Figure 1A,B). Both variables increased in fish fed 10% OLM and upwards compared to the control group, while the number of goblet cells and intraepithelial leukocyte infiltration values did not differ among the different treatments (Figure 1C,D and Figure 2).

No melanomacrophages or liver tissue inflammation were observed in any of the OLM treatments. However, different OLM levels differentially influenced (*p* ≤ 0.05) the degree of fat degeneration (Table 7), and reduced hepatic fat was observed over 10% OLM inclusion.

## 4. Discussion

This study investigates the use of ora-pro-nóbis leaf meal for the production of fish feed for the first time. This plant exhibits high potential in this regard as it is a significant source of nutrients [19] with a nutritionally relevant amino acid composition, especially leucine, phenylalanine, and lysine [27], which may contribute to the production of more economically sustainable and environmentally friendly feeds. The cultivation of unconventional food plants has increased, mainly due to the search for nutritionally rich foods from sustainable agricultural practices.

### 4.1. Growth Performance Variable Assessment

Considering only PFM, WG, DWG, DFI, CYH, CYWH, FY, HSI, VSI, VFI, and SR, the use of OLM as an ingredient in diets for adult Nile tilapia was effective up to a 20% level of inclusion. However, reductions in FCR, PER, and FE from 15% highlight limitations in the efficient utilization of OLM nutrients by Nile tilapia. This may be associated with increased crude fiber content and antinutritional factors, alterations in dietary amino acid profiles due to OLM inclusion, and potential correlations between energy sources. According to Almeida et al. [11], ora-pro-nóbis contains antinutritional factors such as oxalic acid, nitrate, saponins, phenolic compounds, and trypsin inhibitors at the following concentrations: 41.79 mg 100 g^−1^ DM, 16.20 mg 100 g^−1^ DM, 0.29 mg 100 g^−1^ DM, 19.34 mg 100 g^−1^ DM, and 1.82 UTI mg^−1^ DM, respectively. The authors reported no side effects in humans from these concentrations, indicating no contraindications for human consumption. However, no studies concerning the maximum acceptable levels of these elements in Nile tilapia diets are available to date.

Fiber, commonly found in vegetable meals, can negatively impact omnivorous animals, as non-ruminant animals have a limited ability to digest dietary fiber due to their simple gastrointestinal tract [28]. Unlike ruminants, which possess a specialized compartment for microbial fiber fermentation, or herbivorous monogastrics, which present long, voluminous large intestines and sometimes a cecum, other non-ruminant animals do not display such adaptations. In a study with Nile tilapia fingerlings, Hayashi et al. [29] demonstrated that the type of ingested fiber can influence weight gain in this species. Similarly, Meurer et al. [30] reported that increasing crude dietary fiber levels by adding cellulose reduces the retention time of the food bolus in Nile tilapia fingerlings. Consequently, dietary fiber can interfere with nutrient digestion and absorption, i.e., proteins, lipids, and minerals, potentially reducing nutrient utilization efficiency.

Furthermore, OLM is characterized by high protein content but low digestible energy values [19]. The inclusion of dietary OLM resulted in a linear decrease in corn and soybean meal inclusion, accompanied by a linear increase in soybean oil inclusion, maintaining consistent digestible energy levels across experimental diets. This combination of factors likely contributed to improved diet utilization and greater protein efficiency.

### 4.2. Analysis of Body Chemical Composition Variables 

Higher moisture contents in carcasses follow an inversely proportional relationship between ether extract and moisture [31], which is, in turn, associated with dietary composition (Table 2), where corn was the main ingredient replaced by OLM. With this substitution, a gradual increase in the amount of crude dietary fiber was observed and, considering that crude fiber influences the food bolus passage rate [30], fat absorption may have been reduced. Additionally, improved protein efficiency ratios may explain the differences in carcass protein deposition observed herein.

The same, however, was not observed in whole fish and fillets, probably due to the presence of viscera in the whole fish and only muscle in the fillet. Fat deposits that affect carcass quality can be divided into discarded and consumed fat deposits. Discarded fat deposits include visceral fat, located in the abdominal cavity around the digestive tract and accounting for up to 25% of the total body weight, depending on the fish species and status, and subcutaneous fat, distributed throughout the fish’s body, more prominent in dorsal or ventral zones. Ventral subcutaneous fat is one of the belly flap components located in fish abdomens, representing the portion of flesh beneath the ribs [32].

### 4.3. Analysis of Hematological Variables

Hematological parameters are essential in understanding environmental and nutritional animal health impacts and are widely employed in aquaculture. Fish from treatments T5%, T10%, and T20% exhibited lower hemoglobin levels than the control treatment (T0). This may be associated with oxalic acid, an antinutrient found in relatively high concentrations in OLM [11].

Oxalic acid has the ability to form complexes with iron during digestion, a crucial element in hemoglobin composition, resulting in ferrous oxalate. Although this process does not prevent iron absorption, it renders iron unavailable to the exposed organism, leading to its excretion in urine [33]. Reduced hemoglobin concentrations may, thus, indicate a decreased capacity for oxygen transport to tissues [34]. However, it should be noted that the mean hemoglobin values determined herein fall within the normal range for Nile tilapia, of 19.9 to 2.6 g dL^−1^ averaging 7.98 [34]. Although hemoglobin levels can provide insights into fish health, they cannot be employed as a standalone biomarker. In the present study, no significant erythrocyte reductions were observed (*p* > 0.05), although the means decreased numerically, which is understandable given the relationship between red blood cells and hemoglobin.

Leukocytes, blood cells involved in innate and acquired immune responses, were not significantly different among treatments, indicating that OLM did not compromise the Nile tilapia immune system. Similarly, no alterations were observed in the evaluated metabolites and enzymes, whether in plasma or hepatic tissues.

### 4.4. Intestinal Histological Variable Analysis

The significantly higher villi observed in tilapia fed higher OLM levels indicate an ability to adapt to dietary modifications, as longer villi result in greater absorptive process efficiencies [35]. Consequently, increased villi height led to increased villi area, an important nutrient absorption surface. However, OLM is a fiber-rich food that causes low food bolus retention. According to Meurer et al. [30], this may result in reduced nutrient utilization, as the feed spends insufficient time in the digestive tract to undergo adequate digestion and absorption processes. Therefore, the plasticity in increasing the height and area of villi may comprise a way to improve nutrient absorption.

Goblet cells and leukocyte infiltration values were not increased following dietary OLM inclusion. According to Hlophe and Moyo [36], goblet cells produce a mucus that lines the brush border, serving as a lubricant and providing protection against chemical and mechanical damage. An increase in the number of these cells could indicate greater intestinal irritation, which may suggest immune response activation to dietary antinutrients [37]. Therefore, the lack of increased goblet cell numbers and the absence of intraepithelial leukocyte infiltration changes, both common in inflammation areas, further support the absence of negative OLM immune response effects in Nile tilapia.

Ora-pro-nóbis leaf meal is highly nutritious and rich in fiber, proteins, vitamins, minerals, and bioactive compounds [38], all of which play essential roles in enterocyte growth and health. Its dietary fibers have a prebiotic effect, stimulating the production of short-chain fatty acids (SCFAs) by intestinal microbiota, such as butyrate, which promotes cell proliferation and strengthens the intestinal barrier [39]. Moreover, its high protein concentration includes amino acids like glutamine, which is crucial for enterocyte growth and repair [40]. The carotenoids (vitamin A) present in ora-pro-nóbis are indispensable for the differentiation and maintenance of the intestinal epithelium, while zinc contributes to the structural and functional integrity of the mucosa [41,42]. Finally, antioxidant polyphenols help protect enterocytes against oxidative stress and inflammation, optimizing the intestinal environment [43]. Therefore, it is likely that the combination of all these factors contributed to enterocyte growth.

The results reported herein contrast with those reported by Anand et al. [44], who observed shorter villi, an increased number of goblet cells, and microvilli degeneration after including 15% *Sesbania aculeata* leaf meal in the diets of *Cyprinus carpio*. However, they are similar to the findings of Amin et al. [45], who used lower levels (0.005, 0.010, and 0.020%) of *Ziziphus mauritiana* leaf meal in Nile tilapia diets and demonstrated that leaf meal inclusion increased villi height, width, area, and perimeter and that leaf meal improved the growth and intestinal health of the fish through scanning electron microscopy.

The absence of melanomacrophages, as well as tissue inflammation, indicates liver tissue health. In the present study, even the highest level of OLM inclusion (20%) did not cause liver damage. In fact, the effect was positive, as OLM inclusion promoted decreased fat degeneration. These results are similar to those observed by Yu et al. [46], who reported that *Sedum sarmentosum* extract played a hepatoprotective role in Nile tilapia presenting high-fat-diet-induced fatty liver disease. The observed changes suggest that *S. sarmentosum* extract exerts its protective effects by regulating lipid metabolism, steroid biosynthesis, and apoptosis.

It is also important to note that, as the OLM diets investigated herein were formulated to meet the nutritional requirements of Nile tilapia, no intense fatty degeneration (>70%) was observed in fish from any of the treatments, only mild to moderate.

To minimize the effect of probable antinutrients present in OLM, particularly in the 15% and higher inclusion levels, and to optimize its use in aquaculture, further studies are recommended to evaluate the use of ora-pro-nóbis as a feed additive. This plant is rich in soluble and insoluble fibers, as well as antioxidants, and beneficial bacteria in the gut microbiota can ferment these soluble fibers and use them for growth, promoting a healthy gut environment [47]. Although no studies have investigated the use of OLM in fish to date, it is worth noting that preliminary evidence of these effects already exists in humans. Vieira et al. [39], for example, investigated the influence of OLM on the adhesion of probiotics to intestinal epithelial cells in overweight men and found that its consumption improved intestinal health and maintained high adhesion of *Lactobacillus casei* to intestinal cells.

## 5. Conclusions

The inclusion of up to 10% OLM in the diet of Nile tilapia can be recommended without compromising key parameters. This is supported by improvements in feed conversion and protein efficiency rates, alongside growth performance parameters comparable to the control condition. Additionally, the absence of adverse effects on hematimetric and biochemical blood indices highlights this diet’s safety. Furthermore, increased intestinal villus height and area, which enhance nutrient absorption, demonstrate tilapia capacity to adapt to dietary changes. Finally, the more fibrous nature of OLM diets contributed to reduced hepatic fat degeneration, resulting in healthier Nile tilapia livers. These findings collectively highlight the potential of OLM as a viable and sustainable dietary component for Nile tilapia in aquaculture activities.

## Figures and Tables

**Table 1:** Bromatological chemical composition of ora-pro-nóbis leaf meal (OLM) (based on dry matter).

Bromatological Chemical Composition								
	DM%	CP%	EE%	MM%	CF%	NDF%	ADF%	GE (MJ/kg)
OLM	95.56	24.36	4.54	20.6	18.96	42.28	8.91	15.54

DM—dry matter, CP—crude protein, EE—ether extract, MM—mineral matter, CF—crude fiber, NDF—neutral detergent fiber, ADF—acid detergent fiber, GE—gross energy.

**Table 2:** Formulation and bromatological chemical composition of experimental diets containing different levels of ora-pro-nóbis leaf meal, based on natural matter.

	Levels of Ora-Pro-Nóbis Meal				
Ingredients	0	5	10	15	20
Soybean meal	55.25	55.14	55.03	54.92	54.8
Ora-pro-nóbis meal	0.00	5.00	10.00	15.00	20.00
Corn	39.3	33.02	26.74	20.46	14.18
CaHPO_4_	2.25	2.31	2.37	2.43	2.5
Soy oil	2.30	3.67	5.04	6.41	7.78
Vitamin–mineral mixture ^1^	0.50	0.50	0.50	0.50	0.50
Salt	0.10	0.10	0.10	0.10	0.10
BHT ^2^	0.01	0.01	0.01	0.01	0.01
CaCO_3_	0.29	0.25	0.21	0.17	0.13
Analyzed composition (nutrient)					
Moisture	3.53	3.07	2.98	3.11	3.28
Crude protein	30.25	30.51	30.56	30.59	31.03
Digestible protein ^3^	24.00	24.00	24.00	24.00	24.00
Gross energy (MJ/kg)	18.99	18.81	19.55	19.40	19.11
Digestible energy (MJ/kg) ^3^	12.56	12.56	12.56	12.56	12.56
Lipids	5.22	6.18	7.55	9.40	10.95
Mineral matter	6.41	7.02	7.82	8.64	9.49
Starch ^4^	31.46	27.51	23.57	19.62	15.67
Crude fiber	4.12	4.64	5.55	6.30	6.79
NDF ^2^	17.56	20.25	20.22	20.13	23.19
ADF ^2^	4.22	4.98	5.06	4.85	6.67
Lignin	0.65	1.07	1.33	1.02	1.58

^1^ The guaranteed vitamin and mineral supplement levels per kilogram of product were as follows: vit. A = 1,200,000 IU; vit. D3 = 200,000 IU; vit. E = 12,000 mg; vit. K3 = 2400 mg; vit. B1 = 4800 mg; vit. B2 = 4800 mg; vit. B6 = 4000 mg; vit. B12 = 4800 mg; folic acid = 1200 mg; calcium pantothenate = 12,000 mg; vit. C = 48,000 mg; biotin = 48 mg; choline = 65,000 mg; niacin = 24,000 mg; Fe = 10,000 mg; Cu = 6000 mg; Mn = 4000 mg; Zn = 6000 mg; I = 20 mg; Co = 2 mg; and Se = 20 mg. ^2^ BHT—butyl hydroxy toluene, NDF—neutral detergent fiber, ADF—acid detergent fiber. ^3^ Values based on formulation; ^4^ Estimated values based on the percentages of ingredients used.

**Table 3:** Productive growth performance, nutrient utilization and survival of Nile tilapia fed diets containing different levels of ora-pro-nóbis meal inclusion.

	Levels of Inclusion (%)					
Variables	0	5	10	15	20	*p*-Value
IW	297.00 ± 15.00	288.50 ± 13.05	289.17 ± 10.16	297.67 ± 12,95	278.50 ± 11,44	0.22
FW	596.2 ± 49.03	640.5 ± 51.57	627.3 ± 57.82	567.6 ± 17.79	543.1 ± 32.15	0.11
WG	299.2 ± 48.71	352.0 ± 51.72	338.1 ± 47.76	270.0 ± 21.84	264.6 ± 43.23	0.12
DWG	3.05 ± 0.50	3.59 ± 0.53	3.45 ± 0.49	2.75 ± 0.22	2.70 ± 0.44	0.12
DFI	6.07 ± 0.81	6.28 ± 0.66	6.39 ± 0.80	6.19 ± 0.43	6.43 ± 0.18	0.95
FCR	2.22 ± 0.11 ^ab^	1.86 ± 0.16 ^b^	1.96 ±0.11 ^b^	2.39 ± 0.04 ^a^	2.53 ± 0.40 ^a^	0.01
PER	1.72 ± 0.07 ^a^	1.93 ± 0.12 ^a^	1.82 ± 0.04 ^a^	1.50 ± 0.02 ^b^	1.39 ± 0.21 ^b^	0.00
FE	0.50 ± 0.02 ^bc^	0.57 ± 0.03 ^a^	0.54 ± 0.01 ^ab^	0.44 ± 0.01 ^cd^	0.42 ± 0.06 ^d^	0.00
CYH	86.26 ± 0.28	85.42 ± 0.98	86.64 ± 1.58	88.09 ± 1.08	86.69 ± 0.43	0.08
CYWH	56.65 ± 1.06	56.35 ± 0.57	55.69 ± 0.69	55.47 ± 1.56	55.35 ± 2.68	0.79
FY	35.89 ± 1.95	36.61 ± 1.09	35.33 ± 0.67	34.72 ± 2.38	33.79 ± 1.79	0.36
HSI	2.15 ± 0.10	2.12 ± 0.18	2.08 ± 0.20	1.68 ± 0.28	1.93 ± 0.16	0.07
VSI	10.07 ± 0.35	11.17 ± 0.48	10.31 ± 1.74	8.71 ± 1.24	9.64 ± 0.38	0.12
VFI	3.34 ± 1.17	3.76 ± 0.49	2.91 ± 1.38	2.66 ± 0.53	2.09 ± 0.93	0.33
SR (%)	90 ± 1.00	100 ± 0.00	100 ± 0.00	100 ± 0.00	90 ± 0.43	0.37

IW—initial weight (g); FW—final weight (g); WG—weight gain (g); DWG—daily weight gain (g); DFI—daily feed intake (g); FCR—feed conversion ratio; PER—protein efficiency rate; FE—feed efficiency; CYH—carcass yield with head (%); CYWH—carcass yield without head (%); FY—fillet yield; HSI—hepatosomatic index (%); VSI—viscerosomatic index (%); VFI—visceral fat index (%); SR—survival rate (%). Means followed by different letters on the same line differ significantly from each other according to Duncan’s test (*p* ≤ 0.05).

**Table 4:** Chemical composition of Nile tilapia fed diets containing different levels of ora-pro-nóbis meal inclusion.

		Levels of Inclusion (%)					
	Nutrients ^1^	0	5	10	15	20	*p*-Value
Fillet	MO	75.33 ± 1.19	75.75 ± 0.87	75.70 ± 0.60	76.58 ± 0.94	76.58 ± 0.59	0.34
	CP	20.36 ± 0.48	20.52 ± 0.69	20.76 ± 0.38	20.73 ± 0.34	20.74 ± 0.39	0.80
	EE	5.00 ± 0.73	4.31 ± 0.69	4.53 ± 0.31	3.64 ± 0.69	3.95 ± 0.44	0.13
	MM	1.08 ± 0.05	1.10 ± 0.04	1.17 ± 0.06	1.12 ± 0.03	1.11 ± 0.05	0.27
Carcass	MO	68.73 ± 0.88 ^bc^	68.26 ± 0.51 ^c^	68.70 ± 1.32 ^bc^	70.76 ± 0.30 ^a^	70.08 ± 0.48 ^ab^	0.01
	CP	19.23 ± 0.91 ^a^	19.24 ± 0.49 ^a^	18.58 ± 0.40 ^a^	18.38 ± 0.37 ^ab^	17.21 ± 0.88 ^b^	0.02
	EE	10.41 ± 1.10 ^a^	10.24 ± 0.68 ^ab^	10.61 ± 0.89 ^a^	8.70 ± 0.88 ^bc^	8.34 ± 0.59 ^c^	0.02
	MM	4.07 ± 0.45	4.54 ± 0.83	3.99 ± 0.29	4.50 ± 0.04	4.92 ± 0.27	0.16
Fish	MO	66.62 ± 1.06	65.38 ± 0.92	66.31 ± 0.80	67.22 ± 2.50	67.12 ± 0.75	0.51
	CP	16.42 ± 0.73	17.42 ± 2.57	16.19 ± 0.94	16.80 ± 0.79	17.28 ± 0.52	0.75
	EE	11.80 ± 2.14	13.28 ± 1.90	12.43 ± 2.90	10.24 ± 1.46	11.27 ± 0.42	0.43
	MM	4.29 ± 0.18	4.40 ± 0.03	4.18 ± 0.29	4.52 ± 0.60	4.35 ± 0.23	0.76

^1^: MO—moisture; CP—crude protein; EE—ether extract; MM—mineral matter. Means followed by different letters on the same line differ significantly from each other according to Duncan’s test (*p* ≤ 0.05).

**Table 5:** Mean values of hematological variables of tilapia fed diets containing different levels of ora-pro-nóbis meal inclusion.

	Levels of Inclusion (%)					
Variables ^1^	0	5	10	15	20	*p*-Value
Ery (10^6^ μL^−1^)	1.41 ± 0.03	1.20 ± 0.13	1.17 ± 0.15	1.29 ± 0.05	1.20 ± 0.03	0.15
Hb (g dL^−1^)	7.90 ± 0.18 ^a^	7.24 ± 0.26 ^b^	7.24 ± 0.08 ^b^	7.76 ± 0.22 ^a^	7.01 ± 0.08 ^b^	0.00
MCH (pg)	56.25 ± 2.28	61.05 ± 4.32	62.84 ± 7.56	60.12 ± 3.53	58.36 ± 0.69	0.62
Lym (%)	86.65 ± 2.49	90.81 ± 1.13	85.21 ± 4.73	85.30 ± 7.25	85.33 ± 4.01	0.68
Neu (%)	12.75 ± 2.76	8.61 ± 0.83	14.46 ± 4.84	14.00 ± 3.18	13.92 ± 3.85	0.68
Mono (%)	0.45 ± 0.36	0.54 ± 0.28	0.30 ± 0.15	0.52 ± 0.33	0.71 ± 0.23	0.69
Eos (%)	0.03 ± 0.02	0.03 ± 0.03	0.00 ± 0.00	0.00 ± 0.00	0.03 ± 0.02	0.17

^1^: Ery—erythrocytes; Hb—hemoglobin; MCH—mean corpuscular hemoglobin; Lym—lymphocytes; Neu—neutrophils; Mono—monocytes; Eos—eosinophils. Means followed by different letters on the same line differ significantly from each other according to Duncan’s test (*p* ≤ 0.05).

**Table 6:** Mean values of metabolic and enzymatic variables of tilapia fed diets containing different levels of ora-pro-nóbis meal inclusion.

	Levels of Inclusion (%)					
Variables ^1^	0	5	10	15	20	*p*-Value
Plasma						
TPr	4.06 ± 0.56	4.32 ± 0.28	3.92 ± 0.36	4.21 ± 0.46	3.74 ± 0.16	0.43
Alb	1.08 ± 0.10	1.19 ± 0.12	1.11 ± 0.04	1.05 ± 0.03	1.10 ± 0.03	0.28
Creat	0.42 ± 0.08	0.37 ± 0.22	0.27 ± 0.07	0.29 ± 0.13	0.38 ± 0.04	0.59
TG	303.7 ± 29.36	320.6 ± 29.47	308.3 ± 18.80	270.5 ± 21.00	255.9 ± 55.62	0.72
Glc	74.47 ± 20.75	61.75 ± 5.66	63.92 ± 15.03	72.28 ± 17.73	58.10 ± 13.32	0.40
TC	193.1 ± 13.85	186.3 ± 6.16	181.0 ± 15.03	184.9 ± 17.73	191.0 ± 13.32	0.94
Ca	12.86 ± 0.54	12.12 ± 0.30	12.20 ± 0.41	12.42 ± 0.44	12.43 ± 0.92	0.56
ALP	19.92 ± 2.85	16.22 ± 3.03	15.67 ± 2.06	14.39 ± 1.56	15.95 ± 2.62	0.16
Liver						
AST	15.98 ± 4.43	12.68 ± 2.86	19.07 ± 3.22	13.37 ± 1.94	14.15 ± 2.12	0.15
ALT	6.51 ± 2.73	12.72 ± 4.92	7.28 ± 3.87	8.80 ± 4.6	7.40 ± 3.55	0.39

^1^: TPr: total proteins (g dL^−1^); Alb: albumin (g dL^−1^); Creat: creatinine (mg dL^−1^); TG: triglycerides (mg dL^−1^); Glc: glucose (mg dL^−1^); TC: total cholesterol (mg dL^−1^); Ca: calcium (mg dL^−1^); ALP: alkaline phosphatase (UL^−1^); AST: aspartate aminotransferase (U L^−1^); ALT: aspartate aminotransferase (U L^−1^). Means followed by different letters on the same line differ significantly from each other according to Duncan’s test (*p* ≤ 0.05).

**Table 7:** Means of the degree of fat degeneration (FD) observed in cuts (HE at 400*) of Nile tilapia liver fed diets containing different levels of ora-pro-nóbis flour inclusion.

	Levels of Inclusion (%)					
Variable	0	5	10	15	20	*p*-Value
FD	2 ± 0.00 ^a^	1.5 ± 0.41 ^ab^	1.17 ± 0.24 ^b^	1.17 ± 0.24 ^b^	1 ± 0.00 ^b^	0.01

Means followed by different letters on the same line differ significantly from each other according to Duncan’s test (*p* ≤ 0.05).

## Data Availability

The datasets collected/generated during and/or analyzed during the current study are available on reasonable request to the corresponding author.

## References

[1] FAO, IFAD, UNICEF, WFP, WHO (2024). The State of Food Security and Nutrition in the World 2024.

[2] FAO (2022). The State of World Fisheries and Aquaculture 2022: Towards Blue Transformation.

[3] Omitoyin S.A., Orisasona O., Ajani K.E., Omitoyin B.O. (2023). Effect of graded levels of moringa oleifera leaf meal on growth, haematology and serum biochemistry of african catfish *Clarias gariepinus* juveniles. Aceh J. Anim. Sci..

[4] Farhad F.B., Hashem S., Rana K.M.S., Salam M.A. (2023). Growth performance and hematological responses of silver barb (*Barbonymus gonionotus* Bleeker, 1850) fingerlings to dietary blanched moringa (*Moringa oleifera* Lam.) leaf meal as a substitute of soybean meal. Heliyon.

[5] Banik A., Kumar A. (2022). Perspective on utilization of leaf meal as fish feed ingredient for fish in future aquaculture. International Traditional Foods and Sustainable Food Systems Symposium.

[6] Gatlin D.M., Barrows F.T., Brown P., Dabrowski K., Gaylord T.G., Hardy R.W., Herman E., Hu G., Krogdahl Å., Nelson R. (2007). Expanding the utilization of sustainable plant products in aquafeeds: A review. Aquac. Res..

[7] Troell M., Naylor R.L., Metian M., Beveridge M., Tyedmers P.H., Folke C., Arrow K.J., Barrett S., Crépin A.S., Ehrlich P.R. (2014). Does aquaculture add resilience to the global food system?. Proc. Natl. Acad. Sci. USA.

[8] Adeniyi O.V., Olaifa F.E., Emikpe B.O. (2018). Growth performance and nutrient digestibility of *Clarias gariepinus* (Burchell 1822) fed diets fortified with *Tamarindus indica* pulp and leaf meal. Asian Fish. Sci..

[9] Matos É.J.A., Urbinati E.C., Meurer F. (2023). Leaf Meal in Fish Nutrition.

[10] Simonetti M.G., de Fariña L.O., Simonetti K.T.G. (2021). The potential of the ora-pro-nóbis (*Pereskia aculeata* Mill.) in the National School Feeding Program. Res. Soc. Dev..

[11] de Almeida M.E.F., Junqueira A.M.B., Simão A.A., Corrêa A.D. (2014). Chemical characterization of the non-conventional vegetable known as ora-pro-nóbis. Biosci. J..

[12] Sommer M.C., de Araújo Ribeiro P.F., Kaminski T.A. (2022). Obtention and physicochemical characterization of ora-pro-nóbis flour. Braz. J. Health Rev..

[13] Ribeiro Junior W.A., De Paula J.C.B., Soares V.R., Stulzer G.C.G., Rosalem I.B., De Faria R.T. (2021). Propagation of ora-pro-nobis (*Cactaceae*) from cuttings at different concentrations of IBA. AGRO@MBIENTE ON-LINE.

[14] de Miranda Souza M.R., Pereira P.R.G., Pereira R.G.F., de Paiva Barbosa I., Baracat-Pereira M.C. (2020). Protein yield and mineral contents in *Pereskia aculeata* under high-density planting system. Pesqui. Agropecu. Trop..

[15] Souza R.I., Radis A.C., Barbosa J.F. (2020). Replacement of wheat bran by ora-pro-nobis bran (*Pereskia aculeata*) in broiler diets. Cad. Agroecol..

[16] Peixe B.R. (2024). Anuário 2024 PEIXE BR da Piscicultura.

[17] Abo-State H., Hammouda Y., El-Nadi A., Ahmed H. (2014). Evaluation of Feeding Raw Moringa (*Moringa Oleifera* Lam.) Leaves meal in Nile tilapia fingerlings (*Oreochromis niloticus*) Diets. Glob. Vet..

[18] Chen S., Jia Y., Xu W., Peng J., He Y., Sun J., Pan Q., Peng C., Yang J., Chen X. (2020). Effect of *Moringa oleifera* leaf meal supplementation on growth performance, morphological indexes, antioxidant status and resistance to *Streptococcus agalactiae* of Nile tilapia (*Oreochromis niloticus*). Turk. J. Fish. Aquat. Sci..

[19] Matos É.J.A., Zadinelo I.V., Dias P.S., Urbinati E.C., Meurer F. (2025). Apparent digestibility of ora-pro-nobis (*Pereskia aculeata*) leaf meal by *Nile tilapia*. Pesqui. Agropecu. Gaúch.

[20] Carneiro W.F., Colpini L.M.S., de Souza R.C.T., Bombardelli R.A., Balen R.E., Meurer F. (2020). Effect of the digestible protein-energy relationship on the growth performance of Nile tilapia (*Oreochromis niloticus*) fed fishmeal-free diets. Anim. Feed. Sci. Technol..

[21] Latimer G.W., AOAC International (2019). Official Methods of Analysis.

[22] Winthrobe M.M. (1934). Variations in the size and hemoglobin content of erythrocytes in the blood of various vertebrates. Folia Hematol..

[23] Rasband W.S. (2018). ImageJ. https://imagej.net/ij/.

[24] Albinati A.C.L., Moreira E.L.T., Albinati R.C.B., Carvalho J.V., de Lira A.D., Santos G.B., Vidal L.V.O. (2009). Histological biomarkers—Chronic toxicity for roundup in piauçu (*Lophisiolurus alexandri*). Arq. Bras. Med. Vet. Zootec..

[25] Albinati A.C.L., Soares P.C., Albinati R.C.B., Moreira E.L.T., Lira A.D., Carvalho J.V. (2017). Toxicidade do inseticida Tiametoxam para o pacamã (*Lophisiolurus alexandri*). Pesqui. Vet. Bras..

[26] Leonard J.N., Skov P.V. (2022). Capacity for thermal adaptation in Nile tilapia (*Oreochromis niloticus*): Effects on oxygen uptake and ventilation. J. Therm. Biol..

[27] Botrel N., Godoy R.L.O., Madeira N.R., Amaro G.B., Melo R.A.C. (2019). Comparative Study of Protein Composition and Amino Acid Profile in Five Pereskia Clones.

[28] Silva E.G.B., Marinho A.L., Moreira J.A., Novaes L.P., da Silva A.D.L., Mota L.C. (2016). Bran palm giant with exogenous enzymes addition in pig nutrition on growth. Acta Vet. Bras..

[29] Hayashi C., Meurer F., Boscolo W.R., Soares C.M. (2000). Fiber sources in diet performance of Nile tilapia fingerlings (*Oreochromis niloticus*). Acta Sci..

[30] Meurer F., Hayashi C., Boscolo W.R. (2003). Crude fiber for Nile tilapia (*Oreochromis niloticus* L.) fingerlings. Rev. Bras. Zootec..

[31] Hassan A., Aftabuddin, Meena D.K., Puthiyottil M., Das B.K., Sharma A.P. (2024). Clean valorization of distillery industry co-product for fish cage aquaculture: A waste-to-wealth approach. Blue Biotechnol..

[32] Weil C., Lefèvre F., Bugeon J. (2013). Characteristics and metabolism of different adipose tissues in fish. Rev. Fish. Biol. Fish..

[33] Oliveira L.C.S., Kamonseki D.H., Rostelato-Ferreira S. (2017). Determination of oxalic acid levels in different samples of tomato. Nutr. Rev. Nutr. Vigilância Saúde.

[34] Esmaeili N. (2021). Blood Performance: A new formula for fish growth and health. Biology.

[35] Caballero M.J., Izquierdo M.S., Kjørsvik E., Montero D., Socorro J., Fernández A.J., Rosenlund G. (2003). Morphological aspects of intestinal cells from gilthead seabream (*Sparus Aurata*) fed diets containing different lipid sources. Aquaculture.

[36] Hlophe S., Moyo N.A.G. (2014). Replacing fishmeal with kikuyu grass and moringa leaves: Effects on growth, protein digestibility, histological and haematological parameters in *Clarias Gariepinus*. Turk. J. Fish. Aquat. Sci..

[37] Kousoulaki K., Østbye T.-K.K., Krasnov A., Torgersen J.S., Mørkøre T., Sweetman J. (2015). Metabolism, health and fillet nutritional quality in Atlantic salmon (*Salmo salar*) fed diets containing n-3-rich microalgae. J. Nutr. Sci..

[38] Silva N.F.N., Silva S.H., Baron D., Neves I.C.O., Casanova F. (2023). *Pereskia Aculeata* Miller as a novel food source: A review. Foods.

[39] Vieira C.R., da Silva B.P., do Carmo M.A.V., Azevedo L., Nogueira D.A., Martino H.S.D., Silva R.R. (2019). Effect of *Pereskia aculeata* Mill. in vitro and in overweight humans: A randomized controlled trial. J. Food Biochem..

[40] Kim M.-H., Kim H. (2017). The roles of glutamine in the intestine and its implication in intestinal diseases. Int. J. Mol. Sci..

[41] Takeiti C., Antonio G., Motta E., Collares-Queiroz F., Park K. (2009). Nutritive evaluation of non-conventional leafy vegetable (*Pereskia aculeata* Miller). Int. J. Food Sci. Nutr..

[42] Torres T.M.S., Mendiola J.A., Álvarez-Rivera G., Mazzutti S., Ibáñez E., Cifuentes A., Ferreira S.R.S. (2022). Protein valorization from ora-pro-nobis leaves by compressed fluids biorefinery extractions. Innov. Food Sci. Emerg. Technol..

[43] Macedo M.C.C., Silva V.D.M., Serafim M.S.M., Correia V.T.V., Pereira D.T.V., Amante P.R., da Silva A.S.J., Mendonça H.O.P., Augusti R., de Paula A.C.C.F.F. (2023). Elaboration and characterization of *Pereskia aculeate* Miller extracts obtained from multiple ultrasound-assisted extraction conditions. Metabolites.

[44] Anand G., Srivastava P.P., Varghese T., Sahu N.P., Xavier M., Harikriskna V., Prabhakaran A., Kumari P. (2020). Haematological and histoarchitectural alterations in *Cyprinus carpio* (Linnaeus 1758) fed with sesbania leaf meal. J. Environ. Biol..

[45] Amin A., El-Asely A., El-Naby A.S.A., Samir F., El-Ashram A., Sudhakaran R., Dawood M.A.O. (2019). Growth performance, intestinal histomorphology and growth-related gene expression in response to dietary *Ziziphus mauritiana* in Nile Tilapia (*Oreochromis niloticus*). Aquaculture.

[46] Yu K., Huang K., Jiang S., Tang X., Huang X., Sun L., Pang L., Mo C. (2021). Protective function on liver and proteomic analysis of the improvement mechanism of *Sedum sarmentosum* bunge extract on nonalcoholic fatty liver disease in Nile tilapia. Aquaculture.

[47] Dawood M.A.O., Koshio S. (2019). Application of fermentation strategy in aquafeed for sustainable aquaculture. Rev. Aquac..

